# A qualitative exploration of influences on eating behaviour throughout pregnancy

**DOI:** 10.1186/s12884-022-05135-7

**Published:** 2022-12-15

**Authors:** Lauren Rockliffe, Debbie M. Smith, Alexander E. P. Heazell, Sarah Peters

**Affiliations:** 1 Manchester Centre for Health Psychology, School of Health Sciences, Faculty of Biology, Medicine and Health, University of Manchester, M13 9PL Manchester, UK; 2 Maternal and Fetal Health Research Centre, School of Medical Sciences, Faculty of Biology, Medicine and Health, University of Manchester, Manchester, UK; 3 St. Mary’s Hospital, Manchester University Hospitals NHS Foundation Trust, Manchester Academic Health Science Centre, Manchester, UK

**Keywords:** Pregnancy, Eating, Diet, Qualitative research, Interview, Health behaviour

## Abstract

**Background:**

Pregnancy is often conceptualised as a ‘teachable moment’ for health behaviour change. However, it is likely that different stages of pregnancy, and individual antenatal events, provide multiple distinct teachable moments to prompt behaviour change. Whilst previous quantitative research supports this argument, it is unable to provide a full understanding of the nuanced factors influencing eating behaviour. The aim of this study was to explore influences on women’s eating behaviour throughout pregnancy.

**Methods:**

In-depth interviews were conducted online with 25 women who were less than six-months postpartum. Interviews were audio-recorded and transcribed verbatim. Data were analysed thematically.

**Results:**

Five themes were generated from the data that capture influences on women’s eating behaviour throughout pregnancy: ‘The preconceptual self’, ‘A desire for good health’, ‘Retaining control’, ‘Relaxing into pregnancy’, and ‘The lived environment’.

**Conclusion:**

Mid-pregnancy may provide a more salient opportunity for eating behaviour change than other stages of pregnancy. Individual antenatal events, such as the glucose test, can also prompt change. In clinical practice, it will be important to consider the changing barriers and facilitators operating throughout pregnancy, and to match health advice to stages of pregnancy, where possible. Existing models of teachable moments may be improved by considering the dynamic nature of pregnancy, along with the influence of the lived environment, pregnancy symptoms, and past behaviour. These findings provide an enhanced understanding of the diverse influences on women’s eating behaviour throughout pregnancy and provide a direction for how to adapt existing theories to the context of pregnancy.

**Supplementary Information:**

The online version contains supplementary material available at 10.1186/s12884-022-05135-7.

## Introduction

Supporting women to make positive changes to their health behaviour during pregnancy is an important part of antenatal care to optimise maternal and fetal health. Making healthy changes to dietary behaviour in particular, can reduce the risk of developing a myriad of pregnancy-related conditions and complications, such as gestational diabetes, gestational hypertension, preeclampsia, caesarean section, and miscarriage [1, 2]. The benefits of making healthy dietary changes during pregnancy extends beyond this period, as these changes may be maintained post-pregnancy, improving long-term health outcomes for both the mother and child. This is important, as poor diet is a modifiable behavioural risk factor for non-communicable diseases, which has a major financial impact on the budget of the NHS [3–5].

During pregnancy, women’s motivation to improve their health behaviours may increase. For example, some women report a desire to increase their physical activity levels or to improve the quality of their diets [6, 7]. This presents an opportunity for health professionals to deliver health promotion at a time when women may be more receptive to this information. For this reason, pregnancy is often referred to as a ‘teachable moment’ for behaviour change [8].

Teachable moments are thought to occur when a health event increases an individual’s perception of risk or outcome expectations, prompts a strong affective or emotional response, and results in a redefinition of self-concept or social role [8]. This model provides a level of explanation that can be applied to pregnancy, as this is a health event that requires women to consider risk and outcomes in relation to their own health and that of the unborn child, that may provoke strong emotions such as elation or fear, and for nulliparous women in particular, may provoke a change in self-identity as they adopt the new maternal role [9].

The Capability-Opportunity-Motivation Behaviour model (COM-B; [10]) has also been suggested to be a useful tool to understand pregnancy as a teachable moment. Whilst McBride et al.’s [8] model (referred to hereafter as the TM model) incorporates psychological constructs thought to impact upon motivation, self-efficacy and skill acquisition, the COM-B model also considers the impact of capability and opportunity on women’s gestational health behaviours, providing an alternative explanation by which to understand pregnancy as a teachable moment [11].

Pregnancy, as a teachable moment, is often discussed as one static health event (e.g., [9, 12]). However, pregnancy typically lasts up to 42 weeks and is a continually evolving process from both a physiological and psychological perspective. Therefore, it may be the case that multiple events occurring throughout pregnancy act as individual teachable moments, such as confirmation of the pregnancy, feeling fetal movements for the first time, or attending the first antenatal appointment [11].

Different stages of pregnancy may also create more salient opportunities for behaviour change than others [11, 13]. For example, women frequently experience nausea and sickness during the first trimester of pregnancy [14], which according to the COM-B model may reduce their physical capability to eat healthily. As such, the second or third trimesters of pregnancy may afford more effective opportunities for change once physical capability is restored. Furthermore, sense of maternal identity starts to develop around the end of the first trimester for some women, once there is visible evidence of the pregnancy [12]. According to, McBride et al. [8] this may increase motivation to change dietary behaviours due to a redefinition of identify and self-concept at this stage.

A recent longitudinal study from the UK reported that both the COM-B and TM models explained a greater proportion of the variance in eating behaviour during early and late pregnancy, compared to mid-pregnancy and the postnatal period, suggesting that certain stages might afford more salient teachable moments for change, than others [13]. Whilst these findings highlight that women may require alternative support at different stages of their pregnancy, the quantitative approach adopted does not provide scope for a nuanced understanding of the factors influencing behaviour at different stages throughout pregnancy. A qualitative approach can more fully explore women’s experiences through in-depth enquiry.

This study aimed to explore influences on women’s eating behaviour throughout pregnancy, in order to better understand whether certain influences are more salient at particular stages of pregnancy.

## Methods

### Sample and recruitment

One-to-one, in-depth interviews were conducted with women who were less than six months postpartum. Participants were recruited after pregnancy so that they were able to reflect on their entire pregnancy experience. Women were eligible to participate if they were over 18 years old and had received maternity care in the UK for a singleton pregnancy. Women who were advised to change their diet due to high-risk status or had been on a specialist care pathway for a medical diagnosis that required dietary modification (e.g., gestational diabetes, obesity) were ineligible to participate. All eligible women who expressed an interest in taking part were offered an interview.

Participants were recruited online using social media platforms and forums (e.g., Twitter, Facebook, Reddit) (n = 9), through word-of-mouth (n = 7), and by re-contacting eligible participants from a previous research study (n = 9) [13]. A prize draw to win a £100 shopping voucher was offered as an incentive to participate. To ensure ethnic diversity in the sample, a purposive recruitment approach was utilised [15]. Women interested in participating contacted the first author directly, who provided a copy of the participant information sheet and assessed eligibility via email. Participants from a previous study [13] who had provided consent to take part in further research were re-contacted via email after determining eligibility using previously collected data.

### Procedure

Interviews were conducted online using video conferencing software (Zoom) between November 2020 and February 2021. This methodology was deemed most appropriate to explore the topic in-depth, whilst adhering to COVID-19 government restrictions that were in place at the time [16, 17]. All participants provided written consent and completed a demographic survey prior to participating, using an online form. Interviews were audio-recorded and transcribed verbatim.

An interview schedule was used to guide the discussion, which focused on eating behaviour throughout pregnancy (see Supplementary Material). The interview schedule was designed specifically for this study and was developed based on the study aim (i.e., to explore influences on women’s eating behaviour throughout pregnancy), existing literature, and the research team’s expertise. The schedule was comprised of three sections relating to early-, mid-, and late-pregnancy. For each section of the interview participants were asked if they could recollect their eating behaviour and any changes made during each phase of the pregnancy.

**Fig. 1:**
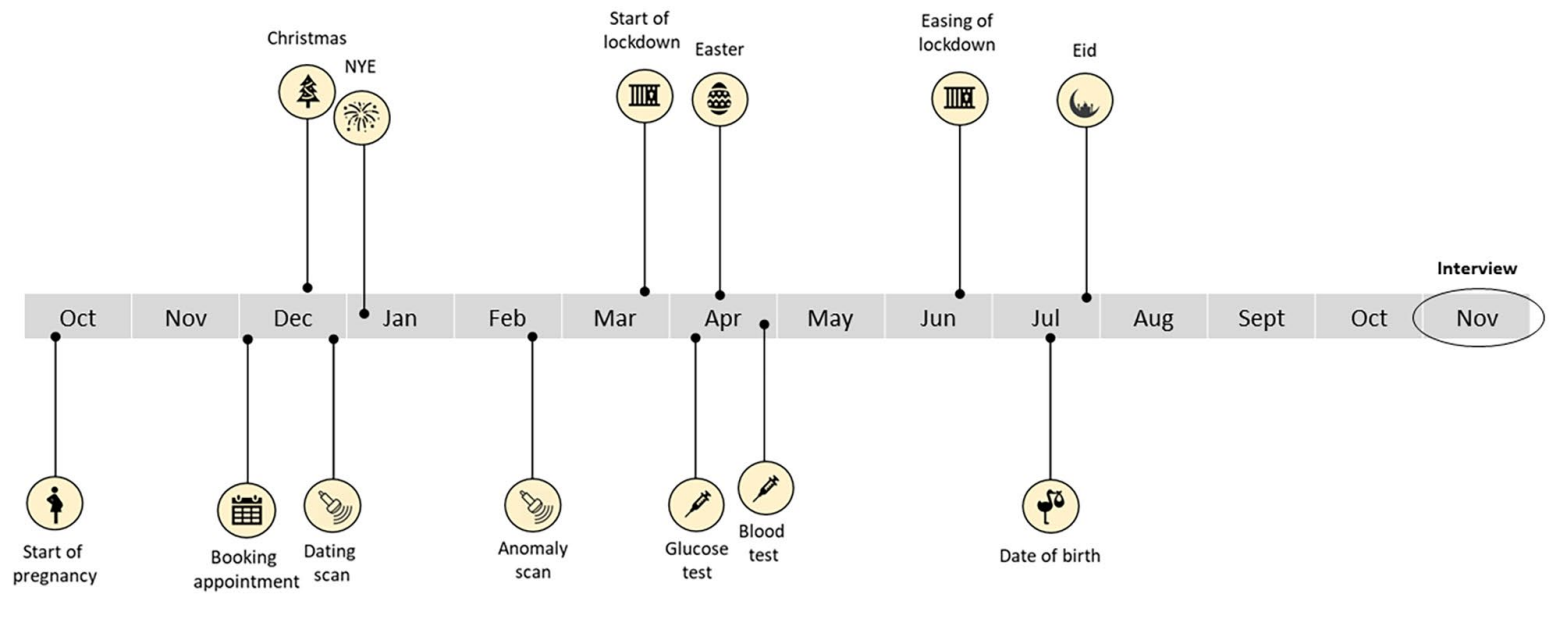
Visual timeline example

To aid participant recall of the stages of their pregnancy, an individualised visual timeline was created in advance of the interviews. The timeline was based on the date the participants gave birth (information which was gathered via the demographic survey) and plotted key antenatal appointments (e.g., booking appointment, glucose test) and events (e.g., religious festivals, COVID-19 lockdowns) that would have occurred during the participant’s pregnancy (see Fig. 1). The timeline was shared with participants using the ‘share screen’ function at three points throughout the interview, to support the interview schedule. This approach borrows from visual elicitation methods, which have been shown to be effective in aiding participant recall [18, 19].

To aid reflexivity, the lead researcher completed a reflective diary entry after each interview and produced a reflective account of the online interviewing process which was submitted for publication [20]. These activities enhanced the interview process by allowing the researcher to reflect on each participant interaction, potential biases and researcher influence, and to consider what went well and how the interviews could further be improved.

All interviews were conducted by the lead researcher, who is a White female non-parent in her mid-thirties. At the time, the researcher was undertaking a PhD, although had previous experience in qualitative methods. No prior relationship was established with participants before the interviews takook place, and no information or characteristics were disclosed about the researcher beyond name and occupation, with the exception of one participant who had been introduced to the researcher by a mutual contact. The wider research team included two Health Psychologists and an Obstetrician, all White British parents with experience of research and clinical care for pregnant women.

Ethical approval was obtained from the University of Manchester Ethics Committee (2020-9584-16317).

### Analysis

Data were analysed thematically according to the six steps outlined by Braun and Clarke [21], using NVivo 12 Plus [22]. As thematic analysis seeks to identify, analyse, report, and interpret patterns within qualitative data [21], it was deemed the most appropriate approach to use. An inductive approach to analysis was taken, whereby themes and interpretations were derived directly from the raw data [23].

In the first phase of the analysis, the lead author familiarised herself with the raw data before applying initial codes, line-by-line, to all data (phase two). These codes were then reviewed and refined. For ease, these codes were initially organised by the phase of pregnancy that they related to. Following discussion and joint interpretation of the data with the research team, the revised codes were then grouped to form thematic categories (phase three) and organised to create a hierarchical thematic ‘map’ (phase four). This thematic map was subsequently revised and reorganised as appropriate, and the theme names defined (phase five). The themes were further refined during the write-up phase of the analysis (phase six).

Analysis of completed interviews was conducted alongside ongoing data collection to allow for refinements to the interview schedule, although no major changes were made, and to assess the richness and sufficiency of the data in relation to the original research question [24]. At the point at which the last interview was conducted, no new key concepts were identified within the data by the lead author, so data collection ceased [25].

## Results

Interviews were conducted with 25 women and lasted an average of 44 min (range 30–60 min). Participants were between 25 and 43 years old (mean = 34) and were between 5 and 24 weeks postpartum (mean = 15 weeks). Participant ethnicities included White British (n = 18, 72%), Other White (n = 3, 12%), Mixed/Multiple ethnic groups (n = 3, 12%), and Asian/Asian British (n = 1, 4%), which is reflective of the population in England and Wales [26]. Most participants were primiparous (n = 14, 56%), married (n = 18, 72%), and in full-time employment either at the time of the interview, or prior to going on maternity leave (n = 15, 60%). Most participants had achieved a postgraduate qualification (n = 16, 64%). Level of deprivation within the sample, as measured using Index of Multiple Deprivation (IMD) quintiles [27], was varied, however most participants (n = 10, 40%) lived in the least deprived areas in the country. Most participants also fell into the healthy weight BMI category (n = 13, 52%) [28]. Participant characteristics are presented in Table 1.

**Table 1:** Participant characteristics (n = 25)

	All participants (n = 25)
Mean age in years (range)	34 (25–43)
Mean weeks postnatal (range)	15 (5–24)
Number of children	
1	14 (56%)
2	10 (40%)
3+	1 (4%)
Ethnic group	
Asian/Asian British	1 (4%)
Mixed/Multiple ethnic groups	3 (12%)
White British	18 (72%)
Other White	3 (12%)
Martial status †	
Single	1 (4%)
In a relationship	5 (20%)
Married	18 (72%)
Employment status	
Full-time	15 (60%)
Part-time	9 (36%)
Unemployed	1 (4%)
Education level	
Postgraduate education	16 (64%)
Higher education	9 (36%)
Levels of neighbourhood deprivation † *	
1 (most deprived 20%)	2 (8%)
2	3 (12%)
3	4 (16%)
4	7 (28%)
5 (least deprived 20%)	3 (12%)
BMI category (kg/m^2^) † **	
Obese (30-39.9)	3 (12%)
Overweight (25 -29.9)	8 (32%)
Healthy weight (18.5–24.9)	13 (52%)

† Total ≠ 25 as data not provided by every participant

*Based on IMD quintiles [27]

**Based on NIHR guidance [28]

**Fig. 2:**
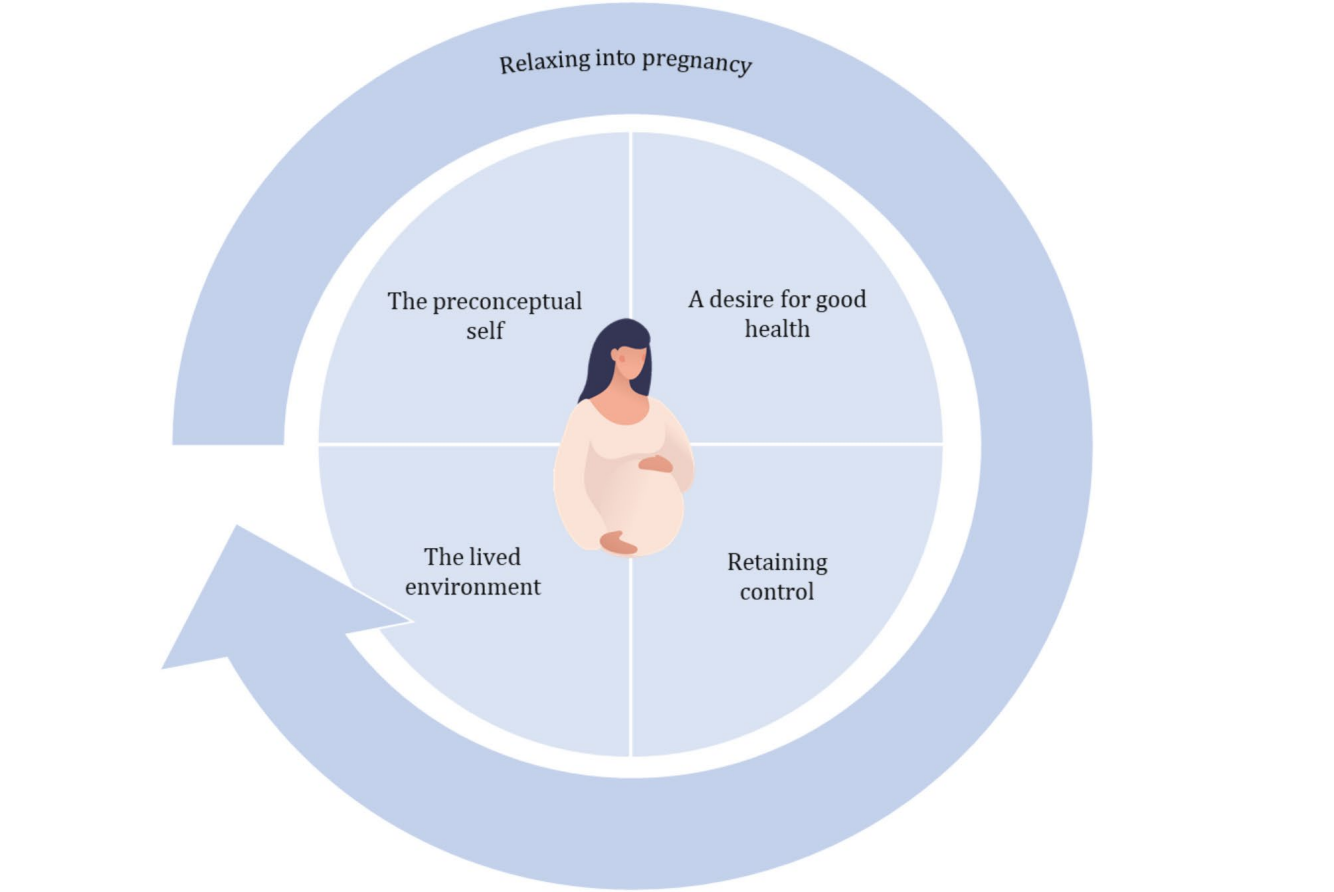
Thematic diagram

Five themes were generated from the data that reflect various factors influencing eating behaviour throughout pregnancy. These themes are entitled ‘The preconceptual self’, ‘A desire for good health’, ‘Retaining control’, ‘Relaxing into pregnancy’, and ‘The lived environment’ (see Fig. 2 for thematic diagram). Participant quotes are presented within the text to illustrate these themes. Barriers and facilitators to healthy eating behaviour identified within the analysis as related to specific stages of pregnancy, have been extracted from the findings and are presented in Table 2.

**Table 2:** Summary of barriers and facilitators to healthy eating behaviour specific to the stages of pregnancy

	Barriers	Facilitators
Early-pregnancy	• Physical symptoms	• Heightened anxiety, especially for those who had previously suffered pregnancy loss or complications
Mid-pregnancy	• Feeling back to normal/reduced physical symptoms • Being visibly pregnant • Eating for two • Receiving healthy scan/test results	• Feeling back to normal/reduced physical symptoms • Increased connectedness with the pregnancy • Receiving healthy scan/test results
Late-pregnancy	• Anxiety surrounding birth plans • Being visibly pregnant • Eating for two • Feeling fed up/the physical burden of pregnancy • Belief baby will not be affected by food choices so late in pregnancy • Receiving healthy scan/test results	• Feeling back to normal/reduced physical symptoms • Concerns about gestational weight gain • Thinking about future postnatal health • Upcoming glucose test • Receiving healthy scan/test results

### The preconceptual self

Throughout pregnancy, women’s decisions about their eating were influenced to some degree by established preconceptual factors. These included their pre-conceptual identity, their previous pregnancy experiences (or lack thereof), and their established eating behaviours.

#### Pre-conceptual identity

Women’s preconceptual identity impacted on the decisions they made about their eating in both positive and negative ways. This included their self-image (e.g., as a *“healthy person”* or a *“thin person”* ^P6^), their occupation (e.g., in a healthcare setting), interests (e.g., science, nutrition), and attitudes, all of which facilitated healthy choices. Cultural heritage also played into some women’s food preferences and choices, which could sometimes lead to less healthy decisions.

*“I’m from a French background, you know. I can bet you my grandmother didn’t give up camembert and red wine when she was pregnant, and her children turned out fine, so [laughs], you know, I’m afraid to say, I indulged a little bit” (P1)*

#### Previous pregnancy experience

The experience of a prior pregnancy could act as both a barrier or a facilitator to healthy eating. Women who had previously experienced undesirable pregnancy outcomes (e.g., surgical delivery due to high birthweight) were often keen to make healthy choices to avoid similar experiences. Likewise, some women were keen to replicate positive pregnancy experiences, such as gaining limited weight, and this also acted to facilitate healthy eating.

Women felt more confident in their ability to manage their diets in subsequent pregnancies. However, reduced anxiety and reduced novelty or excitement surrounding these pregnancies sometimes encouraged a more relaxed approach to eating, including the consumption of ‘unsafe’ foods.

*“I just think I wasn’t as anxious about being pregnant as I was the first time because obviously it’s new the first time you’re doing it and the second time, it’s a typical second child syndrome, isn’t it? Where you’re like, oh – not you don’t care as much but you’re just a bit more relaxed” (P16)*

Multiparous women were keen to model healthy eating behaviours for existing children, and to stay healthy in order to care for them. Consuming sufficient calories to support breastfeeding was also important to those nursing young babies. However, looking after small children could also prevent making healthy choices, particularly when women were time-poor, or using food-related activities to entertain them (e.g., baking).

*“It was things like apple and peanut butter in the day or, you know, throw in some butter with my broccoli at lunch. Things that I’d be happy if he were to say, “Can I have some?” I’d go, yeah. That would have existed before the pregnancy because that’s just part of our family culture” (P8)*

#### Established eating behaviours

The way in which women ate prior to becoming pregnant was typically sustained throughout pregnancy. Women’s food choices were also dictated by pre-existing medical conditions, medical family history, or existing intolerances. Some women reported having problematic relationships with food prior to getting pregnant, owing to pre-existing weight concerns and/or a history of dieting or disordered eating. This sometimes led to increased consciousness and control around food, or alternatively, increased relaxation and unrestrained eating.

*“I think, [it] was maybe tied in a little bit to like previously having an eating disorder and previously having very controlled eating […] There was part of me that, I suppose, felt like, oh, I’m eating for two. You know, this is my chance to eat whatever I want and not worry about it…” (P6)*

### A desire for good health

A desire to support the health of their baby, as well as their own health, was a factor that influenced eating behaviour throughout women’s pregnancies. These desires were often driven by risk perceptions and related concerns, which could result in anxiety and heightened emotions.

#### Health of the baby

The first twelve weeks of pregnancy could be particularly anxiety-inducing, especially for women who had previously suffered pregnancy complications or loss. During this early stage, concerns about nutrition and food safety, and risk of pregnancy loss encouraged women to make positive changes to their eating behaviour. As their pregnancies progressed, women’s concerns often turned to the results of upcoming scans and tests (e.g., the glucose test), which prompted some women to alter their diet by omitting sugary foods.

*“I thought that if I had a week where I didn’t have any kind of chocolate or cakes […] that when I came to do the glucose test I wouldn’t have any of those things in my system” (P15)*

More generally throughout pregnancy, women cited concerns about causing harm to the baby, about the baby’s development, and about pregnancy conditions or complications. This motivated some women to make more healthy food choices and/or to ensure they followed advice and recommendations relating to the consumption of ‘unsafe’ foods. However, the extent to which women adhered to professional advice was influenced by their own risk perceptions.

To further support their baby’s development, women also reported making a conscious effort to eat certain foods for the nutritional properties, or to avoid the development of allergies (e.g., avoiding certain nuts). Women reported increasing consumption of foods such as spinach or red meat to increase iron levels or increasing milk consumption for the calcium content. Similarly, some women were keen to facilitate future breastfeeding by gaining an ‘appropriate’ amount of weight or changing the types of foods eaten to encourage milk production.

*“…I breastfed my first baby for a year. And I had a really good milk supply and I ate porridge every day and I pretty much ate spinach every day as well. And so I was like, I need to start eating my porridge and spinach again” (P10)*

#### Health of the mother

Their own health and wellbeing was also an important consideration for women throughout pregnancy. Dietary changes were sometimes made to relieve fatigue or ease water retention, by consuming additional calories or carbohydrates, or by reducing salt and increasing water intake, respectively. Shielding during the COVID-19 pandemic heightened perceived risk for some women who were motivated to make healthy choices in response. In some cases, unhealthy foods were perceived to serve an important role in supporting personal health.

*“This is something that is maintaining my health [laughs], even though it’s chocolate and, er, pastries and things like that” (P8)*

Concerns about gestational weight gain and postnatal weight retention sometimes encouraged deliberate food restriction. As women progressed into the latter stages of pregnancy, these worries heightened for some, as they became more self-conscious of their growing bodies. At this stage, women also began to think about the impact of their diet on their postnatal health and functioning. For some, a perceived benefit to maintaining a healthy diet in late pregnancy was the potential for a healthy diet to support post-birth recovery, both mentally and physically. Towards the end of their pregnancies, women reported introducing foods into their diet thought to be beneficial for uterine health, such as raspberry leaf tea, or foods thought to help induce labour, such as pineapple or curry.

*“By that stage, I was trying all sorts of things, as you do, eating dates, because they’re supposed to shorten the cervix, and drinking red raspberry leaf tea by the gallon” (P1)*

Women’s mental and emotional health also acted to influence eating behaviour throughout pregnancy. A sense of wellbeing and feeling *“healthy”* ^(P23)^ motivated some women to make heathier choices, whereas negative emotional states (e.g., feeling anxious, stressed, tired, bored) were reported to encourage poorer food choices and ‘comfort eating’. Higher levels of stress and anxiety were reported in early and late pregnancy. In early pregnancy, increased anxiety acted to heighten the protective behaviours of some women (i.e., avoidance of ‘unsafe’ foods). In late pregnancy, anxiety could be trigged by the uncertainty surrounding birth plans due to COVID-19 restrictions. In response, some women reported altering their behaviour, often in an unhealthy direction.

*“… there were points where I had relatives texting me, saying, ‘Are you going to be able to have your home birth? They’ve suspended it in our bit of the country,’ you know. So, the stress of that was certainly a factor in having baked goods around…” (P1)*

### Retaining control

Women perceived a lack of control in varying aspects of their lives throughout their pregnancies. As such, they were keen to retain control over their eating behaviour and as a facet of that, to proactively seek information and reassurance.

#### Control

Whilst women had little influence over the physical changes taking place in their bodies, as well as the changing COVID-19 landscape, maintaining a healthy diet allowed them to retain a sense of control. This was something that endured throughout pregnancy for many women. This desire for control was especially important to those who had previously experienced pregnancy complications or loss, as well as to those who described having a natural inclination to control aspects of their lives. However, for some women this desire to control resulted in restrictive eating behaviours.

*“You can’t control a lot of things in pregnancy. You can’t control, you know, whether you feel sick or you feel tired, but you can control, I guess, your food intake and what’s therefore being transferred to the baby…” (P2)*

#### Seeking information and reassurance

In line with their desire to retain control, some women were keen to make informed decisions about their eating by challenging lay advice and seeking out information themselves. Women reported using sources such as social media, books, podcasts, apps, and websites, to aid their decision-making. However, some women reported having difficulty in obtaining information about culturally specific foods (e.g., traditional Chinese food) or assessing food safety risk while abroad. Confusing or contradictory information sometimes encouraged a more relaxed approach to decision-making about certain foods.

*“…the more I read, the more conflicting advice I found […] I think in the end I just thought, I’m just going to eat my normal healthy diet, it’ll be fine, you know” (P1)*

Women also relied upon information and advice from health professionals (e.g., midwives, mental health professionals) and NHS guidance. The NHS website in particular, was a highly valued resource, above all others. Receiving professional support provided women with reassurance to maintain current eating behaviours (whether healthy or unhealthy), as did receiving healthy scan and test results, although this also motivated positive behaviour change for some.

*“…but for the health and safety kind of things, I do tend to use the NHS because I trust them more” (P12)*

Receiving additional support whilst being under the care of a specialist clinic (e.g., rainbow clinic 1) acted as an additional driver for some women to make improvements to their eating behaviour. However, women reported experiencing a decline in motivation where insufficient professional support or advice was provided. This issue was compounded by the lack of face-to-face contact during lockdown. Some multiparous women believed they received limited support from health professionals, based on the assumption they had existing knowledge from their prior pregnancies.

*“…there was definitely a lack of – a real fall off of motivation because it was just like they [health professionals] don’t even care” (P8)*

### Relaxing into pregnancy

Women’s experience of eating during pregnancy was influenced by their changing physical and emotional state. Faced with physical pregnancy symptoms in early pregnancy, women tried their best to ‘weather the storm’. However, as their pregnancies progressed, many experienced a return to ‘normal’ in terms of their physical symptoms and an increased sense of connectedness and confidence surrounding their pregnancy. This often encouraged women to loosen the reins on their eating behaviour.

#### Weathering the storm

Physical symptoms such as nausea and sickness, were frequently cited as a barrier to healthy eating in early pregnancy. Whilst some women experienced only mild symptoms, others reported feeling out of control during this time, eating only what they were able to *“keep down”* ^(P6)^. Women described being unable to do anything other than surrender to how they were feeling during this time.

*“Yeah, I couldn’t stop – literally, I’d got no control over – I was out of control. Like not like crazy out of control, but I had no control – ‘cos the taste in my mouth, that and the sickness controlled everything, absolutely everything” (P3)*

In navigating this period of pregnancy, women allowed themselves to be guided by their intuition, by listening to their body, and responding to what they perceived to be their needs or those of the baby. The understanding that the symptoms should be transient allowed women to accept the changes to their eating behaviour and relinquish control, although some women reported feelings of guilt at not having been able to consume a nutrient-rich diet.

*“…so yeah, just sort of went into the pregnancy and said, ‘Whatever my body needs to do, my body will do. I’ll go with that and then I’ll sort the damage out after’” (P3)*

Whilst sickness and nausea often improved over time, some women experienced these symptoms throughout the entirety of their pregnancy. Heartburn and fatigue were frequently experienced in the later stages of pregnancy, acting as an additional barrier to healthy eating in mid– to late-pregnancy.

*“So a lot of the choices that I was making between I would have thought maybe twenty-six weeks and thirty weeks, those few weeks, were more so to do with what’s not going to give me heartburn, rather than what’s the healthier option” (P15)*

#### A return to normal

By mid-pregnancy, the severity of physical symptoms lessened for many women, allowing them greater freedom and control over their eating behaviour. Women reported feeling *“back to normal”* ^(P24)^ and most like their pre-pregnancy selves. This could act as both a barrier and a facilitator to healthy eating. In some cases, women described forgetting, or focusing less on their pregnancy, during this stage, which could make it more challenging to make healthy choices. Conversely, increased energy levels during this phase was reported to facilitate home cooking and/or a reduction in snacking. As such, some women took this opportunity to compensate for poor eating behaviours earlier in pregnancy.

*“…I think I allowed myself this time to just sort of get through the first trimester and do what you’ve got to do […] I thought, right, I need to make up for it now and I definitely did make more of an effort to be healthy” (P25)*

#### Connectedness and confidence

As their pregnancies progressed past the 12-week point and into mid-pregnancy, women described feeling more connected with their baby and the idea of being pregnant. This was driven by progressing past what was perceived to be the *“point of no return”* ^(P24)^ with regards to termination, by finding out the sex of the baby, starting to feel the baby moving, and/or becoming visibly pregnant. This enhanced connectedness encouraged some women to modify their eating behaviour in positive ways.

*“Yes, that was the key thing, and I thought, if I get to twenty weeks, you know, he could probably survive. And it wasn’t until then – I mean, I wasn’t silly with my diet, like I said, but it was that scan that made me sort of – made it all real, I suppose” (P19)*

Women’s increased confidence in the viability of their pregnancy was reinforced by healthy antenatal appointments or scans throughout the remainder of the pregnancy. As a result, some women relaxed, or maintained, their eating behaviour, confident in the knowledge their pregnancy was progressing well. As they reached the final stages of pregnancy, this sense of confidence continued to grow, which encouraged some women to become *“complacent”* ^(P23)^ with their eating behaviour.

#### Loosening the reins

Being pregnant acted as justification for some women to relax their attitude towards their food choices, with some women claiming to be *“eating for two”* ^(P6)^, frequently occurring in mid- to late-pregnancy. The development of a visible bump provided justification for increased intake and more indulgent food choices, and served as a visible indicator to others of their pregnant status. Some women believed that any undesirable outcomes related to their unhealthy diet could be rectified in the postnatal period by resuming healthy habits. Compensatory rationales were often used throughout pregnancy to justify unhealthy changes in eating behaviour, based on the principle that these choices did not matter if they had been physically active or made otherwise *“good”* ^(P14)^ food choices. Similarly, food was sometimes used as a replacement for alcohol where women were unable to join in with others who were drinking socially.

Eating behaviours were further relaxed during the latter stages of pregnancy, as women neared their due date. The physical burden of a full-term pregnancy rendered women tired and *“fed up”* ^(P12)^ which lowered their resolve to maintain healthy habits. At that stage in pregnancy, some women believed the baby would no longer be affected by their food choices.

*“…I got to twenty weeks and then loosened the reins a little bit on that. I definitely went through the second half of the second trimester, and the third trimester, was a lot more loose with what I was eating” (P15)*

The desire to relax their eating behaviours was sometimes driven by a desire to embrace their changing body, an experience that was described as *“empowering”* ^(P15)^ and providing a sense of *“freedom”* ^(P15)^ from societal body standards. Learning that their gestational weight gain was not as great as expected also provided reassurance and encouraged the maintenance or further relaxation of their diets.

### The lived environment

Beyond the physical and emotional influences women reported throughout their pregnancy, factors within their lived environment acted to facilitate and hinder healthy eating behaviour, including both the physical environment and norms and judgement experienced in the social environment.

#### The physical environment

Women reported living busy lives whereby convenience foods were sometimes relied upon. The ready availability of unhealthy foods in and around their physical environment encouraged less healthy food choices, particularly whilst at home during lockdown. However, lockdown eliminated the temptation to purchase food during the working day, or to dine out in the evenings, which somewhat restricted the potential for unhealthy food choices. Accordingly, when food establishments re-opened, some women were keen to support local businesses and to take advantage of the government’s ‘eat out to help out’ scheme [30], which sometimes meant making less healthy choices.

*“…working from home there’s always an opportunity to eat. So yeah, I think lockdown very much had an impact on me eating more during pregnancy because it was so readily available” (P21)*

Being at home during lockdown and having a structured routine allowed some women to better focus on being healthy and controlled with the food they prepared. However, food shopping during the pandemic hindered healthy eating intentions where women made rushed decisions whilst visiting the supermarket, if others were doing the food shop on their behalf, or where online shopping made it difficult to check the ‘safety’ of some food items. During this time, food was also reported to be used as an activity in-and-of-itself or a *“treat”* ^(P11)^, and was sometimes used to punctuate points of the working day or week, which could lead to less healthy choices.

*“…[when] I feel like I was at my healthiest was probably during that period because we were eating three times a day, and we were eating probably not so massive portions either…” (P16)*

More generally, seasonal events, such as holidays and religious festivals, were reported to reduce healthy eating. Although the start of a new year encouraged positive changes. The weather was also reported to affect food choices, with hot weather encouraging lighter meal choices (e.g., salads) or reduced intake, and cooler temperatures calling for more *“comforting”* ^(P12)^ meals (e.g., casseroles). The prospect of wearing more revealing clothes in hot weather also motivated healthy eating for some.

#### Social norms and judgement

For many women, their eating behaviour was influenced by the opinions and behaviours of others, which often endured throughout pregnancy. Where those around them supported or encouraged their eating behaviour, whether it be healthy or unhealthy, this could influence food choices. In particular, women’s partner’s eating behaviour, their opinions, and involvement in meal preparation and decision-making, were reported to heavily influence women’s food choices, in both positive and negative ways.

*“… he’s constantly buying me treats, and it’s really nice but also I’m constantly eating the treats because, you know, I’ve not got the willpower to not eat them if they’re there, […] So yeah, that definitely played a part as well in what I ate, I think” (P14)*

The behaviour of other pregnant women was often used as a comparator to their own and to justify food choices. For example, learning that other women had consumed unhealthy diets but gone on to have a healthy pregnancy provided a sense of reassurance for some women about their own diets. Conversely, learning of others’ unhealthy behaviours or negative pregnancy outcomes sometimes acted as a motivator to improve their own behaviour.

*“[I] joined a WhatsApp group with other local mums – and they would post things like, ‘Oh, I’ve had McDonald’s for breakfast’ or ‘I’ve just had a massive Yule log’ and I’m like, oh, I’m okay to have a piece of cake, you know” (P23)*

Knowing inherently that eating unhealthily was *“not the right thing to be doing”* ^(P5)^, some women feared ridicule from family, or judgement from health professionals based on concerns for the baby’s health. Relatedly, some women viewed the lack of social interaction experienced during lockdown, and potential for judgement, as an opportunity to indulge themselves whilst being protected from the gaze of others, should they gain weight. Similarly, for women in early pregnancy, it came as some relief to be at home without work colleagues present to observe unusual eating patterns that might otherwise reveal their pregnant status.

“*When I was nauseous, I would have like – eat crackers to like settle my stomach. And working from home was really good for that… You know, I felt like, because I was at home, I didn’t have to hide that kind of behaviour” (P6)*

## Discussion

This study identified five overarching themes that reflect the different factors influencing women’s eating behaviour throughout pregnancy. These themes capture, at a broad level, influences that are present throughout the entirety of pregnancy. However, there is nuance that reflects barriers and facilitators to healthy eating behaviour specific to certain stages of pregnancy. Moreover, the findings suggest that certain stages of pregnancy create more salient opportunities for behaviour change. For example, sickness and nausea during the early stage of pregnancy is a key barrier to healthy eating, however from mid-pregnancy onwards, a reduction in symptoms and increased sense of connectedness with the pregnancy facilitates healthy change. Therefore, mid-pregnancy provides a window-of-opportunity when women may be more receptive to health promotional advice and are more physically capable of making healthy changes. These findings support previous research which has suggested that behaviour change interventions should be tailored to pregnancy stage to enhance efficacy [13].

Within the data it was also evident that individual antenatal events sometimes motivate healthy changes. For example, women reported altering their diets in advance of their glucose test. This finding supports claims made by Olander et al. [11] who argue that there may be several individual teachable moments that occur throughout a woman’s pregnancy journey. This contrasts with the conceptualisation of pregnancy as one teachable moment in-and-of-itself [9, 12]. However, for some women, receiving healthy test results acted as a barrier to change, as they felt reassured their baby was healthy. This highlights the bidirectional nature of some of the factors identified.

The findings demonstrate that factors influencing behaviour change during pregnancy are nuanced and emphasise the dynamic and changeable nature of pregnancy as a health event, on both a physical and psychological level. Both the COM-B and TM models [8, 10] present heuristics that suggests the mechanisms underpinning behaviour change occur simultaneously, which therefore limits the extent to which these models can be applied within the context of pregnancy [13].

Regardless, the influencing factors identified in the analysis are reflected, to some extent, in the psychological constructs of both models. The COM-B model fully reflects the factors identified in this study, likely due to the fact the model is a general behaviour system designed to be applied to various contexts [10]. However, several factors identified in this study are absent from the TM model heuristic. These include the physical environment and aspects of the social environment, and the influence of pregnancy symptoms. This is consistent with what has previously been reported in the literature [31]. However, the findings from the current study build on this, by also identifying established eating behaviour as additional factor not otherwise reflected in the TM model, which is a facet of women’s preconceptual identity.

The influence of women’s social environment, as identified in this study, may be overlooked in clinical practice. The findings revealed that for many women, eating behaviour was influenced by the opinions and behaviours of others, particularly those of their partner, which is consistent with findings reported elsewhere in the literature [32, 33]. This is important and highlights the need to include partners and/or significant others in conversations surrounding eating behaviour change in clinical practice.

Limited research has explored the influence of preconceptual identity on antenatal health behaviours, however, previous studies have highlighted the importance of identity more generally on eating behaviour. In particular, research has found that viewing oneself as a ‘healthy eater’ can predict healthy eating behaviour [34]–[36]. This is consistent with our findings, which suggested that a ‘healthy eater’ identity facilitated healthy behaviours during pregnancy. Past behaviour and self-identity have also been identified as two key psychological constructs influencing eating behaviour [35, 37] which further supports our findings. Research has also found that past behaviour can predict future eating behaviour, which is in line with our findings that suggest that established eating behaviour and previous pregnancy experiences influenced antenatal eating behaviour [35, 37, 38]. As such, it may be beneficial to support reproductive-aged women to improve their preconceptual health behaviour, using population-level interventions. This approach may subsequently enhance health behaviours during pregnancy by addressing women’s preconceptual identity (e.g., as a ‘healthy eater’) and past behaviour, and potentially improve health outcomes into the postnatal period and beyond.

These findings highlight the role of the ‘self’ in understanding eating behaviour. Whilst the TM model suggests that a redefinition in self-concept or social role is a key psychological mechanism underpinning behavioural change, the findings from this study consider that established identity and behaviour, rather than a redefinition per se, may also play an important role in influencing behaviour. As such, the development of a pregnancy-specific model of behaviour change including a construct that reflects preconceptual identity, or the ‘self’, would be particularly valuable.

### Strengths and limitations

This study provides new insight into the variety of influences on women’s eating behaviour throughout pregnancy, as well as stage-specific influences. However, there are several limitations that should be acknowledged. The participants included in the sample were mainly women from White British backgrounds, all of whom had completed higher or postgraduate education, which may indicate self-selection bias. These similarities in demographic characteristics mean that the findings of this study may not reflect the experiences of women from other backgrounds or identify influencing factors specific to those groups of women. For example, women from Black and Asian ethnic backgrounds report experiencing issues with communication and relationships with healthcare providers [39], which may impact on their willingness to seek support regarding diet and nutrition, as well as limit the scope for health professionals to support behaviour change during routine consultations.

As per the eligibility criteria, the findings from this study do not reflect the experiences of women who were advised to change their diet due to high-risk status or who were on a specialist care pathway for a medical diagnosis that required dietary modification. Factors influencing eating behaviour for women in these groups may be different from those receiving routine care and advice. As such, it would be beneficial for future research to explore whether the factors identified in this study apply to women from these respective groups.

Many of the participants reported that they valued having a healthy diet. It may be the case that social desirability bias affected the way in which participants responded to the interview questions, as healthy eating can be viewed as an external demonstration of ‘good mothering’ [12] and admitting to the contrary may therefore feel socially unacceptable to some women [40, 41]. These altered narratives mean that key influences may have been missed in the current study. Alternatively, self-selection bias may have meant that women with healthier diets were more motivated to participate in a study about eating behaviour. Failing to capture the experiences of women with less healthy diets limits our understanding of the broad range of factors influencing different women. For this reason, it will be important for future work to sample purposively to ensure recruitment of a more diverse sample of women in order to overcome these issues.

An additional consideration is that many of the participants in this study were pregnant throughout lockdown. Women’s pregnancy experiences during this period will be unique, in relation to the practical aspects of their pregnancy (e.g., attending antenatal appointments alone, birth plans), as well as the psychological and emotional aspects [42, 43]. Whilst it is unclear to what extent this will have impacted on the eating behaviour of the participants who took part in this study, other research has reported changes in the dietary intake of pregnant women during this time [44, 45]. It is therefore important to keep this in mind when interpreting the findings.

### Implications for theory and clinical practice

The findings from this study have implications for the delivery and timing of interventions in both research and clinical practice. Whilst much of the clinical guidance in antenatal care fails to consider the impact of timing in health promotion [46], our findings suggest that certain stages of pregnancy, or antenatal events, may provide more opportune moments for health professionals to intervene and deliver health promotional advice. For example, during mid-pregnancy, when women have restored physical capability to eat healthily. Furthermore, maintaining an awareness of women’s preconceptual identity (i.e., understanding existing lifestyles and behaviours) may enable health professionals to better support women. Alternatively, encouraging a change in health identity (e.g., as a ‘healthy eater’) has been suggested to be a potentially effective approach to support women in improving their eating behaviour and sustaining these changes in the long-term [47]. Going forward, enhanced recommendations are necessary to better support health professionals to provide appropriate advice, matched to the different stages of pregnancy.

From a theoretical perspective, the study findings highlight the limitations of both the TM model and COM-B model when applied to the context of pregnancy. The changeable nature of pregnancy and potential for different stages to create more salient teachable moments is at odds with the static view of behaviour change afforded by existing models. Furthermore, the TM model failed to account for several factors identified as influencing eating behaviour. This emphasises the need for the development of an enhanced pregnancy-specific model of behaviour change, that considers the unique and dynamic nature of pregnancy as a health event.

## Conclusion

During pregnancy, certain stages, or antenatal events, provide more salient opportunities for behaviour change, than others. Existing models of teachable moments fail to account for the changeable nature of pregnancy, as well as the influence of the lived environment, pregnancy symptoms, and established eating behaviour. These findings provide a direction for how to adapt existing theory in order to develop an enhanced pregnancy-specific model of behaviour change. In clinical practice, it will be important to consider the different barriers and facilitators presenting throughout the antenatal period and for health promotional advice to be matched to pregnancy stage, where possible.

## Electronic supplementary material

Below is the link to the electronic supplementary material.

Supplementary Material 1

## Data Availability

The datasets generated during and analysed during the current study are not publicly available as participants did not consent to data sharing.
