# Supplementary material for: A qualitative exploration of influences on eating behaviour throughout pregnancy
Source: BMC Pregnancy Childbirth. 2022 Dec 15;22:939. doi: 10.1186/s12884-022-05135-7 (PMC9754306; doi:10.1186/s12884-022-05135-7)
Supplement: Supplementary file 1 — Supplementary Material 1 [file 12884_2022_5135_MOESM1_ESM.docx]

**Interview Schedule**

Introduction

- Introduce self
- Purpose of the study
- Probable length of interview
- Audio-recorded
- Anonymous/confidential
- Can stop at any time/refuse to answer
- Any questions
- Explain focus on eating behaviour in different phases of pregnancy
- If confirmed eating disorder, check comfort with topic

1. Thinking back to the start of your pregnancy, can you tell me a bit about your eating and any changes you made? *refer to timeline*
   - Time/event/gestation?
   - Barriers/facilitators to change?
   - Motivation (for self/baby)
   - Influence of others
   - Information received/needed
   - Practical/environmental factors
2. Thinking back to the middle of your pregnancy, can you tell me a bit about your eating and any changes you made? *refer to timeline*
   - Time/event/gestation?
   - Barriers/facilitators to change?
   - Motivation (for self/baby)
   - Influence of others
   - Information received/needed
   - Practical/environmental factors
3. Thinking back to the end of your pregnancy, can you tell me a bit about your eating and any changes you made? *refer to timeline*
   - Time/event/gestation?
   - Barriers/facilitators to change?
   - Motivation (for self/baby)
   - Influence of others
   - Information received/needed
   - Practical/environmental factors
4. End of interview
   - Any questions or comments on issues not covered in the interview
   - Reassure anonymity/confidentiality
   - Offer copy of findings once study complete
   - Thank for time
